# Post-mortem Researches

**Published:** 1843-04

**Authors:** Bennet Dowler

**Affiliations:** New Orleans


### WESTERN JOURNAL

OF

### MEDICINE AND SURGERY.

---

APRIL, 1843.

---

ART. I.—*Post-mortem Researches.* By BENNET DOWLER, M. D., of New Orleans.

[The reader will excuse the references to dissections not yet published. A want of time to copy from my MSS. in folio volumes must be my apology for using the scissors, instead of the pen, in collecting the detached portions from which I have patched this article into its present form. For a part of the dissection, on which the paper is founded, I am under great obligations for assistance, to the following gentlemen: Dr. Wederstrandt, Resident Physician of the Charity Hospital; Messrs. Saunders and Bates, resident students of the same; Drs. Osborne, Young, Luzenberg, and Nicholson.]

When in the course of medical inquiry, we meet with dogmatism on the one hand, skepticism on the other, and uncertainty every where, we cannot do better than to appeal at once to the decisions of post-mortem anatomy, which, though they may fail in settling some points in dispute, will deceive us in none, and satisfy us in many. One author asserts, that there are one hundred and fifty fevers; another, that there is but one. What Goëthe says of the satirical poet, is applicable to the medical controversialist, who surrounds himself with a "dazzling fence of logic," and is eager for the conflict—"When I have called the bad, *bad*, how much is gained by that? The man who would work aright must not deal in censure, must not trouble himself about what is *bad*, but show and do what is good."

The book of Morbid Anatomy, though written by Nature, is a book of knowledge to those only who read patiently, and interpret its language truly. Pride and self-sufficiency, alone, will deride and decry this modest, safe, though laborious mode of investigation. It was a homely but just saying of one of the reformers, that every man carried a Pope in his belly. There is, however, one panacea for the cure of medical infallibility, which is this: let a practitioner, of this class, carefully dissect fifty or more persons treated by himself, or by another; let him estimate the blunders he has committed—blunders, that an accurate knowledge and a careful examination of the land-marks of disease, might have prevented! and, though his stock of pathological fiction may be diminished by this process, he will lose nothing valuable. Romance will be re-placed by reality. The piratical maxim, "that dead men tell no tales," is erroneous; they are the best witnesses.

To say that morbid anatomy throws no light upon medical treatment, is an admission flattering to empiricism, unjust to our science, fatal to investigation, opposed to truth, and at best, an indirect attempt to excuse professional indolence. Some may think that the following quotation from an able writer is too sanguine in its anticipations; but, what good can we derive from despair? "The time cannot be far off, when the term fever must be entirely discarded from our books, and diseases named according to the tissues which they implicate. Then, and not till then, can it be expected that the laws of deranged action will be properly interpreted, or fully comprehended. All diseases, I feel confident, will ultimately be found to have a local origin and habitation. The artificial nosology of Sauvages, of Hoffmann, Cullen, &c., has had its day." (Prof. Gross' Path. Anat. vol. i, p. 30.)

It is, perhaps, true, that pathological anatomy is in advance of therapeutics. Both are in a state of infancy. The sooner we learn the true position of the enemy, the better.

The sentiment which Boswell makes Johnson utter concerning his opinions, applies but too often to the physician: "I don't like to have any of my opinions attacked. I have made up my faggot, and if you draw out one, you weaken the whole bundle."

Numerical aggregations, numerical analyses, numerical averages, promise results, which, though less certain than mathematics, less poetical than speculation, will, with every succeeding generation, approach nearer to perfection. The amount of labor by this method, for a single individual, would be absolutely appalling to the most ardent imagination; "but many hands, make light work." The book of symptoms, the book treatment, the book of morbid anatomy, remain to be written upon this statistical system. To count correctly, to overlook nothing, to observe philosophically, is a heavy undertaking, more especially as the chaff and the wheat, the ore and the dross, must often of necessity be collected at the same time: the laborer cannot always pre-determine which are the essential pathological facts, anterior to results, since these are the objects of his pursuit, and are not, as yet, supposed to be fully appreciated and known. A few facts, however correct, cannot be relied on for establishing general results, or principles. Analysis is the ordeal which tries his materials, and consumes the uselesss. Even if he discover nothing new, he confirms what was discovered before, and shows its proportional frequency, order, and connexion with other events. The NUMERIC METHOD, is something better than the EASY METHOD, CONJECTURE.

A more extraordinary statement than that of Louis was scarcely ever made, by a cultivator of medicine whose experience had been very extensive: "he says at the close of his labors, when he submitted *all* his facts to the unerring test of arithmetical analysis, *that in every instance were the a-priori conclusions which he had formed from the recollection of his own facts found to be erroneous.*" The import of this statement seems to be, that all systems of medicine, except the ARITHMETICAL, are more likely to prove false than true, upon analytic examination.

In order to test this apparently absurd, and paradoxical principle, I spent considerable time, for several successive days, in examining the tongue alone, in persons in all stages of yellow fever, writing down the appearances at the bed side, in the charity hospital, during the epidemic of the year 1841; and, though I had been extensively engaged in practice during several epidemics, and had written many cases, which I have not to this day analysed, yet I found my previous opinion had been erroneous, so far as the cases above mentioned afford a rule by which to judge. I had supposed, from memory, that the tongue of most persons affected with this fever, had some well marked morbid appearances. But, though some were found to be red, some white, some pointed, some tumid, some dry, &c.; yet, *the majority were natural.* I am far from thinking that memory, when tried by the mere test of arithmetical medicine, will prove generally thus treacherous, or that the cases above mentioned are sufficient to prove, in most cases of yellow fever, that the tongue is healthy.

NUMBER, as an element in working out results, is a safe guide, only so far as its facts are accurate, pertinent, and essential. In morbid anatomy, which is, in connexion with symptoms, the foundation of correct treatment, these are all important, otherwise, we must fall into errors the most fatal under the imposing array of STATISTICS, and MEDICAL MATHEMATICS. AGGREGATIONS may be true, and yet of no value.

The object of this paper is, therefore, to notice some of the *difficulties*, *errors*, and *modifying circumstances*, incidental to a just appreciation of morbid appearances, some of which have been often overlooked or misapplied. The numeric is eminently a false method, when its facts are inaccurately stated.

The *sooner* post-mortem examinations are made, the better. This matter we will illustrate as we proceed. The French writers, who excel in pathological anatomy, have not, certainly, overlooked the great changes that take place in the body within thirty-six hours after death. If they have erred it is in overcharging the picture. But in this, as in some other cases, their knowledge is in advance of their practice. Andral says, that, "in almost every body, opened at about thirty-six hours after death, reddish effusions are found in the cavities, red ecchymosed spots in the great cul-de-sac of the stomach," &c. (Path. Anat., vol. i, p. 49.) And yet, strange to say, this is about the average period, for post-mortem examinations, among the most eminent French pathologists; a fact which throws no little obscurity and doubt, over the results of their numerous observations. Open almost any of their works, and it will be seen, that their autopsies have been delayed from twenty-four to forty-eight hours after death. Louis, in his work on consumption, records fifty-one cases in which the period of the dissection is mentioned, making an aggregate of fifteen hundred and five hours, affording an average to each case of more than thirty hours; his other works, will, I suppose, give about the same average, except his book on the yellow fever of Gibraltar, which gives fifteen.

If I am not much mistaken, the celebrated Broussais *never* mentions the period of his post-mortem examinations at all. In Cook's Morgagni, the same omission is almost constant. Bertin's work on the heart, approved by the Academy of

Medicine, has thirty-three dissections, the average to each being nearly twenty-nine hours after death.

Rilliet and Barthez, on the pneumonia of children at Paris, record a number of examinations, none sooner than twenty-eight hours, and some as late as forty-four hours. In Billard's interesting work on the diseases of children, the period of the numerous dissections is not mentioned with any precision, except in two or three cases. He often says the dissection was performed next day. Now, this may mean seven or eight hours, or it may mean nearly two days. A person dying at 10 o'clock, A. M., may be examined the next day at 5 or 6 o'clock, P. M., forty or forty-one hours after death. Though fractional parts of an hour may be of no importance, even in hot weather, yet, when the dissection is delayed two days, as is often the case with these and other authors, many conflicting elements conspire to efface appearances that existed at death, or to create others, simulating anatomical characters of diseases which did not exist.

Out of one hundred and fourteen yellow fever dissections, not to mention others, a large number were made, or at least begun, from five to ten minutes after breathing ceased, before cadaveric injection, permeation, congestion, and exudation, had time to cause any alteration; hence, it may be inferred that the exact condition of the organs is truly reflected.

I have caused the body to be placed on its right side, for example, immediately after death, both in the night, when the air was cool, and in the day, when it was warm, and in from three to six hours, have observed that the upper or left side was comparatively bloodless, while the lower was loaded with blood externally and internally; the intestines being finely injected, and much reddened, in such dependent parts as were most favorable for the influence of gravitation. (Dissection XLIV.) But dead bodies differ very much from each other in their susceptibilities to cadaveric infiltration. DEATH, in the common acceptation, may, and often does, take place hours before the body dies in a physiological sense. Volition, thought, sensation, respiration, and the heart's action have ceased, and for all the purposes of existence, life is no more; but it does not hence follow, that all the vital actions are extinct; both animal and organic life may exist in many cases. As a proof of this, muscular contraction and capillary circulation, which these dissections fully establish, might be mentioned.

As MUSCULAR CONTRACTION, after apparent death, is not known to exert a modifying agency over the morbid appearances, it is referred to here, merely as confirming the doctrine of capillary circulation, as surviving that of the heart and large arteries—a doctrine, which if true, renders tardy dissection a most fallacious guide in judging of venous congestions, vascular turgescence, organic reddenings, blanchings, &c.

The precise boundary line between life and death, is dubious and undefined, and the dissector cannot commit a greater blunder than to wait for the work of putrefaction, or even post-mortem infiltration, gravitation, &c.

According to my observations, muscular contractions, after death, are more common in the yellow fever subject than in any other class of fever victims.

A slight scratch with the back of the knife across the course of the muscular fibres, in any part of the body, causes the muscles to contract, and to swell into firm ridges exactly at the line of mechanical irritation. The summits of these elevations are sometimes an inch above the surrounding level, with bases of two or three inches in diameter, the muscular fibres assuming zigzag, wavy lines, until they relax into repose again.

Observe the arm as it lies extended upon the table, supple and relaxed—we strike it with a horn, a cane, or metallic rod, a conductor or non-conductor of electricity, either on the skin or on the naked muscle—contraction takes place instantly, or sometimes slowly: for example, we strike the biceps muscle, about its middle—the arm is bent, the hand, perhaps, strikes a violent slap against the face, or rises more slowly with an uniform motion pointing to the zenith, and then gradually falls back from the perpendicular to the horizontal position. This operation may be repeated for several hours, in some cases just so long as the muscular contractility remains unimpaired. In some subjects, this property does not exist, and it varies in different parts of the same subject; the masseter muscles lock the jaws firmly together, in some cases before death, oftener just after; the recti and other abdominal muscles, in a few instances, become rigid in like manner, while the fore arm bounces from the table after every blow given even by the fist of the operator. When the rigidity of the entire muscular system exists, this property is lost; but where the entire body is supple, it does not follow that the power of post-mortem contraction is always present. It exists in the fibres of the heart, but is seldom sufficient to move the whole organ.

Examples might be multiplied were it desirable: the following are supposed sufficient for the present occasion:

E. M——: (Dissection XCII.) A stroke on the arm between the shoulder and elbow, caused the fore arm to rise from the table, and after continuing perpendicular about a quarter of a minute, it slowly fell back to the horizontal position. During the period that the arm continued elevated, the muscles where the blow had been given were contracted into a ridge, which, gradually sinking, the arm fell down to the level again. These experiments were often repeated with the same results; other muscles, in different parts of the body, jerked or contracted when scratched or irritated.

O. S——: (Dissection XCIV.) This subject when struck, as in the last case, contracted his arm strongly, sometimes holding it up a minute after the stroke. The experiment was repeated for several hours.

Not wishing to enter upon the subject of the capillary circulation, only so far as it may exert a modifying influence over the post-mortem appearances, I may however be permitted to say the following facts prove that the direct action of the heart, and its indirect, or suction power, joined to the suction produced by respiration, are not necessary to the rapid motion of the venous blood.

It has been observed by dissectors, that, in the dead body the left side of the heart and all the great arteries are generally found empty. Now of all conceivable facts, this is precisely the fact which ought not to happen, upon the theories which are usually brought forward to explain the capillary circulation. We might, on the contrary, expect to find the left side of the heart and arteries distended, the veins empty. It is erroneous to say that the heart and arteries possess a contractile power, which, after death, expels the blood. We have but to look into the body to see the arteries, not collapsed but hollow, not contracted but large, not gorged with blood during the last moments, when the heart is no longer able to impel it onward. Considering the emptiness of the arteries, it is surprising that they do not contract or collapse completely when exposed to the pressure of the atmosphere, without counteracting distention from blood within their calibres. Moreover, the circulation of chyle by the lacteals, and of lymph by the lymphatics, cannot be accounted for by the heart's action, directly or indirectly.

I had not proceeded far in the yellow fever dissections, in 1841, before several cases of rapid and copious hæmorrhage took place from incisions in the scalp for the purpose of removing the skull-cap, and from incisions in other parts of the body. The quantity of the blood, the force with which it was discharged, and the elevated points from which it flowed, all indicated a power greater than gravitation as well as essentially different in its mode of operation.

F—— P——, aged nineteen, was, about fifteen minutes after death from yellow fever, placed on an inclined plane, the head being the highest part of the body. An incision, beginning an inch above the eyes, and continuing around the head, at the same distance above the ears, discharged, from the forehead, temples, and other parts, one pound and a quarter of blood, in about fifteen minutes: on removing the skull-cap and dura mater, without wounding the brain, the same quantity flowed in half an hour, natural in color, coagulating as in health. (Dissection XXI.)

The body of a man aged thirty, two hours after death from yellow fever, was placed upon the dissecting table, the head being the most elevated part; a circular incision, made an inch above the ears, discharged in half an hour from sixteen to eighteen ounces of good colored blood, which formed a coagulum of good consistence. (Dissection XX.)

A young man, ten minutes after death from yellow fever, was placed on a table, as above; the brain was quickly removed, and in a few minutes after, about twenty-four ounces of blood flowed from the vessels of the head. The blood was of a good color and coagulated firmly. (Dissection XVIII.)

L. B——, aged twenty-one, immediately after death was placed on the dissecting table, the body being very warm and the veins full: the chest was quickly opened. Dr. Young, of this city, who assisted in the dissection, thought he discovered a slight movement or contraction of the heart. The subclavian vein was opened at about the middle of its course. From the orifice the blood shot up two or three inches, an elevation greater than any part of the body, running with such force as to appear like a vital action. From three to four pounds of good colored blood, clotting as it cooled, were discharged in about half an hour. (Dissection LVII.)

G. J——, of Pittsburgh, aged thirty-two, in one hour after death, discharged from an orifice in each jugular, made by Mr. Saunders, student of medicine, eight pounds of healthy blood, according to the estimation of several medical gentlemen. At first the blood shot out some distance. In the second and third hours after opening the veins, about twenty ounces more flowed, before opening any of the cavities. The muscles, which were of extraordinary size and beauty, possessed contractility for several hours. (Dissection LXVI.)

In the case of J—— B——, of L———, the post-mortem blood-letting was nearly as profuse as in the above. (Dissection LXII.)

J—— C——, aged thirty-four, about five minutes after death presented the following evidences of capillary circulation:

The veins of the neck and extremities were prominent. A vein was opened in the left arm—the blood flowed copiously, and jetted freely, on moving the muscles. On passing a ligature around the right arm, as for ordinary blood-letting, the veins became more prominent; one being opened with a lancet, the blood shot out as in the living subject to the distance perhaps of eighteen inches. The left jugular was immediately opened, from which the blood flowed freely, but without jetting; and in an instant after, the abdomen and chest were exposed, by a rapid dissection. One of the branches of the coronary vein of the heart, upon its most elevated part, was punctured, and though this twig was not larger than several others which were distributed upon the hearts' surface, yet, in a few minutes, (ten to fifteen,) it discharged one pound or more of blood. The omenta, mesentery and other viscera, had their external veins beautifully and equally distended. The hollow veins being cut, from three to four pounds of blood accumulated in the cavities, forming good clots.

Without anticipating the appearances of the brain in this case, I may remark, that it was examined last, and though the veins were numerous and large in its envelopes, yet they were nearly empty, owing, probably, to the previous discharges by the decending cava. (Dissection LXXI.)

E. M——. About an hour after death, the veins being distended, blood-letting was performed in the arm, in the usual manner, but without a ligature: the stream was of good size, and shot up, falling in an arch of four or five inches in diameter at first, but, gradually diminishing, it ran down the skin of the arm, amounting in about thirty minutes to nearly as many ounces, though the arm was elevated above any other part of the body—it was also lowered—the abdomen and chest were opened—the heart was pressed; but, all these changes produced none upon the stream, which, becoming weaker and weaker, at length ceased. The right side of the heart was greatly distended; and the coronary veins upon the highest surface of the heart, when punctured, shot out blood freely. This subject retained the power of muscular contraction for hours after death. (Dissection XCII.)

O. S——. On incising the scalp, the blood ran chiefly from the forehead and temples, in a double stream, uniting into one, upon the back of the head, nearly as large as a goose-quill, and in a few minutes, amounted to about one pound: on removing the skull, without wounding the brain or its inner envelopes, a like quantity was discharged, with still greater rapidity. It happened, accidentally, during dissection, that the ligature and compress were removed from the orifice of the vein in the arm, from which, five days before, he had been bled—the blood spouted forth spontaneously, at first in a good stream, gradually diminishing to a slow dropping in thirty minutes. The orifice was un-united, and had a dark color which extended to the surrounding tissue, not less than two inches.

In this subject, muscular contraction continued for several hours. (Dissection XCIV.)

The following case, by some called yellow fever, by some congestive, but which perhaps is not referable to either, is very remarkable:

J—— M——, died, from a fever which lasted twelve days and a half, and was carried directly to the dissecting table—every part of the body was, as far as I could judge, without a thermometer, hotter than in health, appearing to my touch, nearly as hot as in *solar asphyxia*. The febrile smell was very offensive, as in the living body, in the most malignant fevers. The skin was very white, the tongue moist and pale. The external veins, especially those of the arms, were distended, as in health after exercise. Two were opened. The blood flowed rapidly, projecting several inches, and, afterwards, more than a foot, by the aids of compression and the movement of the muscles of the arm. As the blood cooled, a coagulum formed, having a consistent fibrinous, semi-transparent coat. The cavas discharged about four pounds of blood. There was no cadaveric hyperæmia. The anterior, as well as the posterior walls, and viscera of the cavities, were alike supplied with blood. The brain, which was examined last, had but little blood, though its vessels were much developed, dilated, and moderately distended with air bubbles, like the effervescence of soda-water or soap-suds.

About one hour after the body was placed on the table, a free, warm sweat appeared, first, in drops upon the face and neck, and then, extended to all parts of the body. The weather was mild and pleasant. The cadaveric heat, the hot copious perspiration, and the external venous distention, afford strong contrasts to the condition of the living body during congestive fever.

Had this body remained unopened one or two days, what would have become of all this blood? To say nothing of cadaveric injection, and exudations, the transporting power of the capillaries might have deposited the blood in new situations, sponging it out from structures that had suffered during life from acute hyperæmia, engorging others that had been healthy; blanching the former, reddening the latter. At the right side of the heart, venous stagnation probably begins, and its vessels being wholly passive, it travels outwardly towards the extremities of the veins; the capillary power is soon neutralized by an opposing mechanical force, or ended by the entire cessation of vital action.

Since CADAVERIC GRAVITATION aggravates some morbid appearances, removes others, and may observe or change all; and since the capillary circulation may survive the heart's action, and thus, being no longer balanced by supplies from the central fountain, may, in a few minutes after death, remove morbid accumulations, depositing them in the great veins or elsewhere, surely it is important to make post-mortem examinations as soon as possible.

Unusual and protracted HEAT of the body after death, is, I am inclined to think, a presumptive proof that the capillaries are more or less active at the time.

My reason for dwelling so long upon this subject, is, that pathologists have not, so far as I know, adverted to it with a view to apply it to post-mortem appearances. Its physiological bearings do not fall within my design at present, and though I am not able to appreciate, fully and exactly, its modifying agency over morbid anatomy, yet others may be more successful in its application. Facts have not been sufficiently numerous for exact analysis.

Another fact which exerts a modifying power over morbid appearances, a fact which has been too generally omitted by authors, is the following: If the body be warm, the blood fluid, a morbid congestion in one cavity may be removed, or lessened, at least, in some cases, by severing the blood vessels leading to another, especially after an acute disease of short duration. Let the jugulars, or the cavas be cut, or the heart and lungs be removed; in an hour, or less, the brain and the abdomen may become more or less bloodless. Though the blood vessels may have been previously engorged, and their calibers dilated, now, they may appear in a very different condition, collapsed, and comparatively exsanguious. Again, let us suppose, that in two persons recently dead, we proceed to examine whether hepatic congestions exist. In the one case we open the abdomen; we penetrate the liver with the finger, or open its texture with the knife, severing its vessels; the blood wells up rapidly, several pounds may flow in a few minutes; engorgement seems to be present; and yet this happens when there is no congestion at all. In the other case, let all the great vessels be divided—let the liver remain in its natural situation, and let it be the last organ examined, and it will probably be found nearly bloodless; even its parenchyma may not indicate the previous congestion. Without regarding these circumstances, the former liver would be congested; the latter, bloodless or anemic.

COLD, alone, may perhaps remove morbid appearances, and create mechanical congestions, by unequally condensing the solids and fluids of such parts as undergo refrigeration the most rapidly—congestions resembling morbid turgescency. Cold, acting on the superficial capillaries, and other vessels, may force the blood towards the centres, engorging the warmer, and more dilatable parts: even in the living body, cold, more particularly congelation, causes paleness or bloodlessness upon the surface, and of course sanguineous centralisation.

Difficulties, in morbid anatomy, result from our limited knowledge of the exact appearances of each structure, proper to the HEALTHY ANATOMY. Death, from almost any cause, whether from decapitation, hanging, drowning, asphyxia, or fire-arms, may change the healthy appearances, instantly, in many textures. The rarest dissections are those of persons, who have died from accidents, not connected with acute or chronic diseases. The natural vascularity of a part must be estimated, in order to judge whether there be an acquired or morbid increase of vessels: so of color, consistence, and magnitude.

In order to acquire just ideas of this branch of the inquiry, comparative anatomy is generally available. Some easy lessons may be had of the butcher, at the slaughter house, where different animals are killed, by different methods.

Over-nice distinctions which indicate no palpable differences, that millinary of morbid anatomy, retard and discourage the student.

For example, there has been much dispute about the precise color of the mucous tissue of the stomach. This organ is one of the most accessible, the easiest examined, one that has occupied beyond all others the attention of investigators; yet, there exists great difference of opinion, about the healthy appearance of its internal coat. "It has been described as being white, greyish-white, reddish, greyish approaching to yellow and red, straw-colored, &c. Billard, in whose opinion Dr. Hodgkin is inclined to place most confidence, states it to be *a dead milky white.* According to Buisson and Bichat, the color is a deep red, and according to Sabatier and Habicot it is of a reddish purple and deep purple. Gavard, Boyer, Sœmmering, Chaussier, and Adelon, make it of variable shades between red and gray. Rosseau, who derived his opinion from the examination of the bodies of criminals dying by the executioner (by the gullotine we presume), states that the color of the gastro-intestinal canal *is white,* or rather faintly tinged with red. We are ourselves rather disposed to agree with H. Cloquet who describes the usual appearance of the membrane as being of a *reddish-white* and *mottled—comme marbrée.*"—(British and For. Med. Rev., April, 1841.)

The COLOR of the internal structures should be noted, soon after their exposure to the air. This is a matter of much importance, for in a few minutes after the removal of an organ from the body, it becomes red or roseate, owing not to disease, but probably to its imbibing oxygen gas. This acquired color is superficial, and by making a fresh incision into the solid viscera as the liver, lungs, spleen, brain, &c., some hours after exposure, the original color, as at death, will be observed without material alteration. After the frosts of October, 1841, this coloration appeared to take place with great celerity, and often it assumed a scarlet hue which might readily be mistaken for a morbid appearance, by one not duly considering the circumstance to which I allude.

Simple coloration, unaccompanied with structural changes, being so variable, is of small value, and is, at the same time, one of the principal sources of erroneous deduction in morbid anatomy. In not a few diseases, the lesions account for the cause of death, nearly as well as decapitation. It is better, in doubtful and unexplained cases, to acknowledge our ignorance at once, than indirectly to dodge the question, by refering it to slight discolorations, especially such as happen to the mucous tissues of the air-passages, stomach, &c., which, still more than the skin, are liable to be blanched, or spotted with reddish, purplish, and dark hues, from many different causes, besides the last agony. Seldom, if ever, do we see *vascular turgescence* or *redness* of the arachnoid, in the most violent form of the disease called *arachnitis*, while nothing scarcely is more common than *opacity*, *thickening*, *serosity*, *sub-arachnoid* infiltrations, and especially fibrinous or lymphy exudations: the latter product, Professor Gross says, "is invariably of inflammatory origin (without our being able to detect the *slightest redness*, &c.)—a law than which none is more satisfactorily established in pathological science." (Path. Anat. vol. i, p. 59.)

Cadaveric coloration, is the source of innumerable errors in those dissections, where that convenient, theoretical, and summary word *inflammation*, is vaguely used and much patronised. This term is like a sieve for carrying water. It may be necessary, in theraputics, as indicating a certain class of medicinal agents, but is not necessary in morbid descriptions, *mere descriptions.* In the dissection of those white, non-vascular, firm indurations, called scirrhus, I have had some experience, and still more in their treatment. In about one half of these degenerations of the mouth and neck of the womb, the recovery has been complete, by antiphlogistics, as absolute diet, position, local irritants, iodine, mercury and emaciating medicines, the residue ending in ulceration, and death. If the treatment furnish any guide, in judging what is, and what is not, inflammation, it would seem that this *white inflammation* offers an excellent type or starting point; the redness often found around white indurations may be accidental. But *whiteness*, *redness*, *blackness*, &c., do not become important unless all the circumstances of the case be regarded. In acute fevers in this city, the intestines, particularly the cœcum, colon, and rectum, are often as *white* as the breast of a boiled chicken, or as prepared tripe; and still oftener *contracted* to a round firm cord, scarcely larger than the thumb of the subject, and for several feet, so completely closed that the French intestinal scissors cannot be introduced without much force; the whiteness and contraction being the only alterations of the parts. Blackness of the intestines may indicate mortification, or nothing morbid at all beyond mere *dyeing*, as if it were a piece of cloth; black vomit filling the bowels will sometimes, in from ten to twenty hours after death, *dye* all the tissues of the bowels black. Blood, according to its accidental hues, will in like manner dye them red, claret, mahogany, &c., without any changes of consistence whatever.

Mamelonation in its usual and most simple state, is a change, the pathological import of which is but little understood; though, as a matter of fact, it deserves to be noted. It consists of eminences much greater than those on the surface of the roughest orange, alternating with depressed lines upon the internal surface of the stomach, usually of a pale white color, though sometimes appearing like red granulations, at least such are its appearances in the victims of fever in this city. It seldom happens. Perhaps a kind of coagulating or constringing power in the gastric juice, or the spasmodic and irregular contractions of those strong, hair-like fibres and vessels, which compose the net work of the sub-mucous tissue, may give rise to this lesion, if such it may be called. Cold causes the skin to assume a slight mamelonation, called "goose-flesh;" alum, gall nuts, &c., causes the mucous membrane of the mouth to contract and form irregular prominences, apparent fissures, &c. Simple mamelonation, without changes of color, cohesion, vascularity, firmness, &c., is, in morbid anatomy, probably far less important than some imagine.

The quantity of morbid fluids is often great in yellow fever, causing much distention of some organs. B——— C—— (Dissection XXXVIII), female, of medium size, had, five minutes after death, besides six or seven ounces of cerebral serosity and indications of copious hæmorrhages from the uterus and vagina, about thirty ounces of blood effused in the pleural cavities, sixteen ounces of blood in the large intestines, and fifty-six ounces of intensely colored, heavy black-vomit in the small intestines, in all, about one hundred and eight or nine ounces: this coloring matter, in a few hours (to say nothing of a day or two), would have spread obscurity over the morbid appearances of the surrounding textures.

Cohesion, one of the most important and certain tests in morbid anatomy, becomes a fallacious guide, under some circumstances, chiefly from the effects of delay. If, for example, the muscles have lost their firmness and natural color and are becoming puffy, &c., what are we to expect but loss of cohesion in other textures, to say nothing of the action of the gastric juice upon the gastro-intestinal mucous tissue? The medico-legal testimony and pathological deductions, based on post-mortem examinations made many days after death, must in many cases be involved in great uncertainty. I may be pardoned for introducing here the following case as illustrative of the *progress of decomposition* in the various organs in this climate, inasmuch as it is interesting, I trust, in another aspect—showing that the stomach may be ruptured spontaneously without any previous indisposition.

It is proper to remark, in explanation of a circumstance alluded to in the following case, that while travellers facetiously call New Orleans, "the wet grave," its cemeteries are really such, excepting those which are constructed with vaults, costing from sixty-five to one hundred and thirty dollars each; the other cemeteries are situated a mile or more from the river, at the termination of the inclined plane, looking towards the swamps, in a dead level, the bottom of the grave being at about the same height as the low-water mark of the river, which circumstance, in connexion with heavy rains and the impossibility of drainage, keeps the graves full of water, so that bailing out a grave is necessary just before inhumation; and even then the funeral service which says, "dust to dust, earth to earth," sounds oddly, since the defunct is usually committed to the water. In the following case the weather, the position, &c., formed an exception, the bottom of the grave was made in firm, tenacious mud, and contained a stratum of water about two inches deep. Had this body been exposed to the air, instead of the humid grave, decomposition would have been greatly retarded. An opinion has prevailed, even in this city, that animal decomposition is unusually rapid in our climate. The contrary is the fact, unless humidity be present. Humidity in this city is sometimes great and sudden, unconnected with rain, amounting almost to a shower within doors, owing to the fact, that the north and the south winds often meet suddenly face to face; a rapid condensation of moisture follows, without fogs however. But more generally dessication (from a powerful sun and brisk wind) prevails at least during autumn. We have extremes, but the drying property of our air is very remarkable, and to this cause I attribute the slowness of putrefaction which I have observed so often, even in hot weather. It appears that in some instances where moisture is excluded, the body does not putrefy at all. Several years since while engaged in collecting materials for the *grave-yard statistics of this city*, the sexton of the Catholic cemetery informed me that on opening a vault in the upper tier he found a body, long since entombed, completely dried or dessicated, without any signs of previous putrefaction: the hair and whiskers firm; the eyes and face though dried were of a natural appearance. Similar facts have been related by travellers in the east. "We observed, (says Captain Lyon in his travels in North Africa 1818–'19–'20,) many skeletons of animals which had died of fatigue on the desert, and occasionally the grave of some human being. All these bodies were so dried by the heat of the sun, that putrefaction appears not to have taken place after death. In recently expired animals, I could not perceive the slightest offensive smell; and in those long dead, the skin with the hair remained unbroken and perfect, though brittle." (Lyells' Geology, p. 119.)

Having been summoned by the coroner of the Parish of Jefferson, to make a post-mortem examination, before a jury of inquest, at the McDonald cemetery opposite New Orleans, a mile from the river, I proceeded to the place of disinterment, at 12 o'clock, M., October 1st, 1842; the weather being dry and moderately warm.

Previous History. H—— J——, a mulatto (whom I had attended some years before for gun-shot wound), aged thirty-five, resident in New Orleans for twenty years, stout, bony, muscular, with but little adipose tissue, but always healthy—workman in the foundry at Gretna—at 8 o'clock, A. M., September 28th, 1842, after working as usual, ate his breakfast of beef-steak, potatoes, bread and coffee, abundantly—left the table, walked ten paces, sat down, placed his hand over his stomach, knelt down, said he was "devilish sick," the only words he spoke; his pulse became imperceptible, his skin cold; he was dead, fifteen minutes after finishing his meal; was buried the same day; and, by the order of the Judge, was disinterred for inquest seventy-six hours after death. Before reaching the coffin, cadaveric gas became intolerably offensive. The coffin was large, strong and apparently tight; the corpse, which was much enveloped in cloths—the head in bandages—filled the entire coffin, except that part corresponding to the legs. The coffin contained about one gallon of liquid, supposed to be an exudation from the body, being less fluid than water, having an oily, bloody, and turbid appearance; some of it had leaked out.

Corpse swollen, puffy; universal gaseous inflation of the cutaneous, cellular, adipose and muscular tissues doubling the size of the body, not excepting the extremities; integuments of the face and head from two to three inches thick, lips, ears, and eyelids, four inches; the neck, three or four feet in circumference; the scrotum, nearly two; the penis, one; muscles, bloodless and softened; great abdominal convexity from gaseous distention; foam issuing from the mouth and nostrils.

*Head:* hair loose; pericranium and dura-mater non-adherent, without much loss of tenacity; arachnoid and pia mater softened; brain like molasses or pus in consistence, being of a dirty gray or white color, with sanguineous tints in dependent parts; this description applies to the upper portion of the spinal marrow; the vertebræ were separable by the hand alone.

*Chest:* lungs, cohesion moderately diminished, color, gray, with redness in dependent parts, being greatly collapsed, occupying about one-sixth of the cavities; mucous tissues of the air passages reddish; bloody effusion, mixed with serosity, about four pounds; pericardium puffy; heart thin, flabby, pale, nearly white, totally empty (as were its great vessels), and though its muscular tissue was effervescing with minute air bubbles, it was free from elasticity, being only about one fourth of the usual size; aorta, pleuræ, &c., nearly natural.

*Abdomen:* intestines distended, pale, bloodless, not tearing without considerable force, being moderately softened; the mucous tissue pulpy, and free from arborisations, &c.; the bile had infiltrated the subjacent tissues; the gall bladder empty, its coats puffy from gas; the liver was nearly natural in cohesion, elasticity and color; the stomach, small and attenuated, was *ruptured* about four inches along its lesser curvature, it contained about a pint of semi-fluid food; about the same quantity had escaped through the orifice (which was somewhat irregular, though longitudinal), and lay on the left side of the spine near the diaphragm, resting on a clot of blood as large as the hand. Here the examination stopped; other organs not dissected.

A few weeks before this occurrence I met with a case of *rupture of the stomach* in the person of T—— L——, aged sixteen years. (Dissection LXXVII.)

I may be allowed to remark that I never had any personal experience of the *injurious effects* of *animal effluvia in dissection*, except in the above instance. I have known some, who, though good operators on the living, have become sick or faint from dissection of the dead, even when unattended with any strong odors. In the case of J——, just related, a depressing nausea was produced, somewhat ike sea-sickness, which continued for twenty-four hours. Pain in the axilla, nocturnal fever, and tedious pustulations do arise from the smallest wound or scratch in dissection, the dangers of which, however, have been greatly exaggerated, if I might speak from what I have seen.

Gaseous distention of the tissues, though generally the effect of incipient decomposition, is not always so. I have seen it in five minutes after deaths the most rapid in bodies that appeared as free from all the appearances of putrefaction as if they had died from a ball through the brain. It appears in the sub-mucous tissue of the stomach, in the blood vessels of the brain, in the cellular tissue of the neck, and in the heart.

In pathological anatomy, still more than in geology, difficulties arise in the chronological classification of strata; primary, secondary, and recent morbid formations, being often mixed and confused, their *eras* are uncertain.

As in weights and measures, so in healthy and morbid anatomy, *standards* of comparison are desirable. In these dissections, I have often in the absence of any known standards, endeavored to introduce some, though not very precise. Thus, when I speak of the *tenacity* of the arachnoid and pia mater, I mean that they may be peeled from the hemisphere and its depression entire, or nearly so, and that a strip raised on the handle of a knife from one to two inches in breadth, will be strong enough generally to suspend the half or the whole brain without rupturing.

With respect to hypertrophy or enlargement of the organs, they are compared with recollections and with the best descriptions of their size in a healthy state; thus, it is said, their dimensions are augmented one-third, one-half, twice, &c. All this is very inexact; precision is impossible. In different individuals the organs differ in size, in the healthy state. But is not such a standard better than none?

With respect to several of the *hollow viscera*, particularly the stomach, considerable thickness and contraction, or thinness and distention, may exist without necessarily implying any morbid change. An empty stomach is generally very thick, its mucous surface wrinkled, and full of fold or plaits; on the contrary, the presence of much fluid is attended with attenuations of its coats. By inverting this organ, and filling it with air or water, its sub-mucous tissue may be infiltrated by pressure, so as to become one or two inches thick resembling ecchymosis, oedema, emphysema, &c. This tissue is the seat of those arborizations, which have been so often erroneously ascribed to the mucous membrane.

I have not seen in this membrane well-marked vascularity that is, a net work of continuous trunks and branches, as in the conjunctiva, serous membrane, and other tissues; when the sub-mucous tissue has suffered denudations from loss of its mucous membrane by ulceration, softening or otherwise, or when this latter is more or less transparent, arborizations may appear; they are in fact, often almost naked, surviving the wreck of the disorganized tissues to which they had belonged. However red, hard, and swollen the mucous membrane may be, by disintegrating it gradually by scraping, the blood appears in minute points or dots, widening as you progress outwardly, appearing like cones with their apices pointing towards the centre of the stomach, their bases resting in the outer half of the sub-mucous tissue, where a layer of vascularity spreads over the inner surface of the muscular coat in acute hyperæmias of this organ.

It is a very great error, in a scientific point of view, whether in public or private practice, to *pick* or *select* subjects for dissection, on account of something extraordinary in the previous history or in the external post-mortem anatomy, for by so doing we keep among the exceptions, not the general rules; we study monstrosities, not the common run of cases. Few, indeed, can expect out of the irregular to establish its regular laws, as St. Hilaire has done in monstrosity itself.

As a modifier of post-mortem appearances, *treatment* is, doubtless, a very important subject of investigation; a treatment essentially wrong, aggravates, seldom creates the morbid appearances, while the proper treatment lessens their natural tendency more or less. For example, whatever good or bad effects quinine may have in yellow fever and other fevers, given in doses ranging from one grain to two drachms, it appears not to create certain morbid appearances, which some ascribe to it instead of the malady. Serosity of the brain, opacity and tenacity of the arachnoid—vascularity, thickness, infiltration, and firmness of the pia mater, acute hyperæmia of the stomach, &c., are not attributable to that mode of treatment. The candid Eclectic will need humility and patience when he comes to compare and count organs with the Quininian, the Sanguinarian, the Inertiarian, the Hydrargian, the Non-medicationist of the *Method Expéctante*, and other exclusives. In these dissections, to which I have so often alluded, nearly *every mode of treatment is represented and reflected:* in some, *no treatment,* or one wholly palliative was adopted. When a physician, wedded to a particular school of practice, "who has made up and expressed an opinion" in regard to his late, but now defunct patient, comes to the examination and bears testimony against himself, he is doing more than any court in christendom exacts under oath. Rochefoucault says, "we need not be much concerned about such faults as we have the courage to own." A man who dissects none but those who have died under his own peculiar treatment, to say nothing of the biases of pride and theory, must of necessity take a one-sided view, calculated to foster that narrow Idealism which is, in medicine at least, a concentrated impersonation of all that is Real, since it opens the veins, blackens the body with leeches, marches into our vitals with the strongest preparations, compatible and incompatible, that chemistry can produce.

Prof. Graves. of Dublin, strongly insists upon the necessity of morbid anatomy to correct these narrow views of the mere symptom-hunters. "Medicine has followed medicine, each symptom has been the object of attack, until death approaches with accelerated step, and charitably closes a scene distressing to humanity and disgraceful to the cause—I was going to say—of science; but who will give so ennobling a name to this pseudo-practical knowledge—this worse than absolute ignorance. I am not combating phantoms; I do not Quixote like contend with imaginary giants; no, what I have described exists; the picture I have drawn has many an original."

Theoretical words lead to latitudinarian and erroneous results, and should be excluded from dissections altogether.

*The physical appearances* alone must be given—*accurate descriptions,* as in the science of geography, the *morbid and healthy geography of organs.*

I have, in these descriptions, avoided the word *inflammation,* a word that even now is so vaguely applied, as to include the most opposite appearances in color, consistency and size. When an organ is *blanched, reddened, thickened, thinned, softened, indurated, brittle, tenacious, tumefied, ulcerated, puffy, excavated, infiltrated, perforated, enlarged, diminished or otherwise altered,* the fact is so stated, together with all its accompanying *physical characteristics.* This mode, though not very summary and compendious, is in conformity with the dictates of common sense. Let not the matters of fact be confounded with the matters of opinion.

Let us illustrate this matter:—S—— B. E——, of Massachusetts, aged 19, who died of yellow fever, August 5th, 1842, the first fatal case in the epidemic of the season, and who was dissected seventy-five minutes afterwards (by Drs. Wederstrandt, Fenner, Slade, Saunders, and myself—Dissection LXXIII), presented the following appearances of the mucous membrane of the stomach:—"One-third of the mucous membrane, and of the sub-mucous tissue next to the cardiac orifice, were of a deep claret color; when scraped, innumerable small bloody points appeared, without arborizations; the entire membrane having a natural consistence, thickness, and tenacity, peeling in strips an inch long, leaving the muscular coat natural (dull white), except a reddish spot, corresponding to a button-shaped clot of blood, as large as a dime, which formed a dense infiltration of the sub-mucous tissue, with the filaments of which it was intersected." Now, with the exception of this ecchymosed spot, and the intense coloration, all the coats were healthy, free from thickening, puffiness, softening, ulceration, induration, or other departure from the natural state. Here (omitting the button-shaped extravasation, often met with,) we have one element of inflammation. But, is *color, per se,* an indubitable proof of the presence of inflammation, in the absence of all structural alterations whatever? One pathologist may call the above appearance inflammation of the stomach; another may deny this as positively, or doubt as to its pathological character. What must an honest dissector do in the premises? Let him describe the *physical appearances* truly, instead of deciding the pathological question, by writing down the word inflammation. Stronger cases might be multiplied, showing that slate-color and even deep black (sometimes in the form of a very fine, dust-like, powdered charcoal imbedded in the mucous tissue)—arterial, venous, claret, mahogany and other colors, exist independent of any appreciable changes of structure.

The word *congestion*, in its application to the brain, is very often misunderstood, so as to imply a physical impossibility. If the brain when the circulation is natural, be a plenum, or nearly so, as it is admitted to be, and is like water, incompressible, then general congestion, from an absolute augmentation of blood in the cranial cavity, cannot happen unless the bones be separated: the relative situation of the blood changes, not its quantity. The meningeal artery is by a blow ruptured—a broad clot extends over a considerable portion of the brain; a blood vessel spontaneously ruptures in the substance of the brain (apoplexy), forming an irregular coagulum as large as the fist; or, serosity accumulates to the extent of half a pound: but such accumulations in one part must exclude or displace just as much blood from entering the brain to other parts during life. I have seen more than a hundred instances of serosity of the brain, with vascularity of the pia mater; but if the serosity be great, usually the blood vessels are collapsed. So of that doubtful lesion called *hypertrophy of the brain:* if any real enlargement happen, it is probably at the expense of the general circulation, by excluding from the arteries and veins, great and small, an equal quantity of blood.

Many of the French pathologists have adopted a *nomenclature*, in many instances very convenient, and expressive of general conditions or states of morbid alteration; not much is gained, however, by adopting such words as *acute hyperæ-*

*mia, chronic hyperæmia,* as substitutes for *acute* and *chronic inflammation.* In order to avoid endless repetitions, I have been compelled to write the word *healthy or natural,* immediately after the organ mentioned, without describing the characteristics of healthy anatomy, particularly in relation to some of the less important structures, as the lymphatics, pancreas, kidneys, bladder, &c., though *in all doubtful cases the description is given.**

The average TIME required to make a dissection with care, including the time for pencil notes, and then copying, is about four hours. It is true that in the ordinary mode of proceeding, the stomach, &., may be seen in as many minutes. But it is quite a different affair, to examine, to remove, and then re-examine each organ, opening every inch of the bowels, washing, macerating, holding up in the sun every part, &c. Louis says, "practice in order to collect observations, is a *trade,* and like all other trades must be learned, and cannot be divined." A *theoretical abandon* is proper in making a dissection; after it has been faithfully recorded, let it be as completely forgotten as possible while making the next.

The laborious, and to some, repulsive character of these necessary studies, should not deter the conscientious student; for though he should not increase his gold thereby, yet he will *deserve it honestly;* the difficulties incidental to these pursuits, in thinly inhabited country situations are almost insurmountable, and therefore a resort to cities for this purpose is desirable before settling down for life. For this purpose *New Orleans* is unsurpassed by any city in the Union. Here, there is no prejudice against dissections. Her great Charity Hospital offers practical facilities on the largest scale. Every young physician in the south and west, may also furnish himself (without cost) all necessary anatomical preparations.

The DEAD-HOUSE, situated in the rear of the Charity Hos-

* If the average number of healthy organs in each case of dissection amount to twenty, then two hundred cases will amount to four thousand instances, requiring as many special descriptions, unless we adopt the word healthy or natural, in order to avoid repetitions.

pital, in a noisless and unfrequented spot, and consequently favorable for post-mortem investigations, is divided into two apartments, called the reception and the dissecting rooms. In the former the corpse is deposited on a stone floor, by two men whose business it is to coffin the dead. The bodies of males are covered with a sheet only, which is removed before the corpse is placed in the coffin; females are always furnished with garments in which they are buried. If, as rarely happens, the friends of the deceased wish to bury the body, a ticket is placed over its breast by order of the clerk of the Hospital. The dissecting room is ventilated, sky-lighted, and supplied with tables and water. How different from the agitation, noise, confusion, and hurry, incidental to post-mortem examinations in private practice! Here, the operator is not dismayed by the fear that each sound of the saw or hammer, will be echoed back in a sob or a shriek by some relative of the dead, in the adjoining room.

In the dead-house, Europe, Asia, Africa, and America are all represented. The Caucasian or European face, fair and symmetrical, is by far the most common; the Irish, German, French and English greatly predominating, the Mongolian or olive Asiatic, and the African, being most infrequent.

Here, in yellow-fever seasons, the mountaineer of Switzerland, the low-lander of Holland, the Swede from the icy circle, the Mexican from the tropics, the New Englander from his blue hills, and the country Creole from his black swamps, the virgin just arrived at womanhood, and the matron who has not yet passed the meridian of life, lie, side by side, with the saddest features, as if reproaching us for not saving them from an untimely grave. In this contemplation of the dead body upon a large scale, where many, the young, the robust—victims of a single night—lie crowded together, one naturally is reminded of what Moliére makes Beraldo, in the *Maladie Imaginaire*, say: "I do not know a more pleasant piece of mummery, or any thing more ridiculous than for one man to undertake to cure another."

The dead-house, it must be confessed, produces a little skepticism, but it is a wholesome skepticism when making an exploration into the realms of death; even systematic anatomy will abate something of its pretensions on the score of oracularity. The observer finds *irregularities* of structure accumulating on his hands, with surprise, unless he belong to that class, that nothing medical can surprise. One of the greatest anatomists denies that the testicle is ever missing or single, yet I have met with such a case; also a man with organs of generation unmarked with any sign of puberty, being in the infant state of development; a woman with one kidney; in another it was wrong side up; the stomach adherent to the uterus, and irregularities too numerous to mention. One irregularity, referable to morbid anatomy, I will here mention at the risk of being suspected of leaning towards the marvellous. Depending on memory alone (not having leisure at this moment to make an exact estimate), I suppose that about ten per centum of those dying of fevers in this city, have from one to four complete *intus-susceptions* in the small intestines, sometimes six inches in extent, at least such has been one of the results of my observations.

I append the following analysis of the *color of the liver* in yellow-fever, not that it is equal to many other appearances of this and other organs, in pathological importance, but, because it happens to be the most convenient. For though I am collecting facts on fevers and bowel-complaints, and have copied many into the anatomical portion, I have not analysed them with precision. However, I am sure there is no material error in this enumeration. The terms and comparisons used are more or less varied in the original.

| | |
|---|---|
| Yellow, as orange, lemon, straw, brass, gingerbread, cork, - - - - - - - - - | 54 |
| Yellow and milky, - - - - - - | 3 |
| Nutmeg and straw-yellow, - - - - - | 4 |
| Brown and yellow, - - - - - - | 2 |
| Brown and yellow, with lines or streaks, - - - | 2 |
| Yellow, milky, and mahogany, with lines, - - | 1 |
| Yellow and healthy, mixed, - - - - - | 1 |

| | |
|---|---|
| Bronze and reddish, - - - - - - - - - | 4 |
| Pale brown and mahogany, - - - - - - - | 9 |
| Purplish and brown, - - - - - - - - - | 1 |
| Mahogany and white, - - - - - - - - - | 3 |
| Chocolate, - - - - - - - - - - - | 4 |
| Flaxseed, - - - - - - - - - - - | 5 |
| Dark, - - - - - - - - - - - - | 1 |
| Reddish, brown (nearly healthy), - - - - - | 1 |
| Black and brown, - - - - - - - - - | 2 |
| Dark, mustard, - - - - - - - - - - | 1 |
| Black and purple, - - - - - - - - - | 1 |
| Greenish and chocolate, - - - - - - - | 1 |
| Dark and greenish (stratified), - - - - - | 2 |
| Lead-color, - - - - - - - - - - - | 1 |
| Purple and white, - - - - - - - - - | 1 |
| Accidental omissions (in making notes), - - - | 4 |
| Healthy or natural (that is, brown, with slight reddish tint externally, and a yellowish tint internally), - - - - - - - - - | 6 |
| Total examinations (yellow fever), | 114* |

The liver presents many other points for analysis, as to size, cohesion, membranes, anæmia, hyperæmia, &c.; also, oyster-like degenerations of the gall-bladder, effusions of albumen into that cavity, and other important alterations.

* These researches, so far as mere *color* of the liver is concerned, agree in many instances, with those of Louis on the yellow fever of Gibraltar, in 1828; but more generally they are very dissimilar, especially upon the brain, hæmorhages, &c., a fact admitting of satisfactory explanation, without calling in question the accuracy of the commission (of which Louis was one), sent by the French Government to Gibraltar, in 1828, for the purpose of investigating this malady. Their post-mortem examinations were, I believe, nearly all made in the winter, when the epidemic had declined, or nearly so. Except in a single case, they did not, personally, observe the ante-mortem or previous history, and, among their 23 or 24 cases they mention a number as not being yellow fever, as the 13th, 15th, 18th and 24th, and, perhaps, some others. (I quote from memory.) Besides, the yellow fever of Gibraltar and that of New Orleans may differ from each other.

I have not, in this paper, even glanced at the morbid appearances of the FLUIDS. The stupendous conclusions at which certain pathologists seem to have suddenly arrived, appear defective in one essential matter, *evidence, facts.* Even oxygenation, carbonization, de-fibrination, super-fibrination, &c., are better known than appreciated in morbid anatomy. But the new *chemical metaphysics* by which the "alkaloids of opium," "quinine," &c., become chemically combined or converted, or transformed into brain and nervous matter, and then cure chemically by diminishing oxygen, &c., are quite too transcendental for a poor dissector, though well enough adapted to flourish in the realms of German idealism, where the genuine *Monades* are said to exist, out of which it is gravely declared a god will some day be created, if one do not exist already.

With the words, black blood! carbonized blood! putrescent blood! still ringing in our ears, we begin to hear of blood too rich, too fibrinous, over charged with oxygen—(the sensible appearances lean a little towards the latter)—but the easy mode of setting aside the utility of dissections, by referring diseases to the nervous system unaccompanied with any alterations of structure, is an explanation requiring explanation: and so of the fluids. I would like to see the humoralist that can distinguish, by sensible appearances, the blood of a yellow-fever patient from that of the most healthy man; yet they are not always alike.

January, 1843.

